# Anterior tibial artery entrapment syndrome: case report

**DOI:** 10.1590/1677-5449.010017

**Published:** 2018

**Authors:** Marcio Miyamotto, Leandro Castro, Gabrielle Simões Marcusso, Bruna Zimmerman Angelo, Danielle Corrêa de Andrade, Izara Castro de Souza, Ricardo César Rocha Moreira

**Affiliations:** 1 Pontifícia Universidade Católica do Paraná – PUC-PR, Hospital Universitário Cajuru – HUC, Serviço de Cirurgia Vascular e Endovascular, Curitiba, PR, Brasil.; 2 Instituto VESSEL de Aperfeiçoamento Endovascular de Curitiba, Curitiba, PR, Brasil.; 3 Hospital Nossa Senhora das Graças – HNSG, Serviço de Cirurgia Vascular e Endovascular Elias Abrão, Curitiba, PR, Brasil.; 4 Pontifícia Universidade Católica do Paraná – PUC-PR, Hospital Universitário Cajuru – HUC, Liga Acadêmica de Medicina Vascular – LAMEV, Curitiba, PR, Brasil.

**Keywords:** anterior tibial artery, intermittent claudication, arterial compression

## Abstract

Lower extremity intermittent claudication is usually related to atherosclerotic disease. The most common non-atherosclerotic causes are arterites, chronic compartmental syndrome, bone compression, and popliteal entrapment syndrome. The authors report a case of a patient with intermittent claudication related to anterior tibial artery entrapment caused by the interosseous membrane. Magnetic resonance angiography showed compression of the anterior tibial artery during dynamic maneuvers and the patient was managed by releasing the cause of compression, resulting in relief from claudication.

## INTRODUCTION

 Obstructive peripheral disease of atherosclerotic etiology is the most common cause of intermittent claudication, responsible for more than 90% of cases. However, in younger patients who do not have risk factors for atherosclerotic disease, it is essential to investigate other possible causes, such as extrinsic compression of arteries by soft tissues, as in popliteal artery entrapment syndrome, chronic compartment syndrome, compressions caused by bone abnormalities, and arterites. 1 ^-^ 3

 Non-atherosclerotic obstructions are generally caused by compressions related to popliteal artery entrapment syndrome, resulting in a typical presentation of intermittent claudication of the legs, known as spastic claudication. Entrapment of other vessels in the leg is rare, and there are few reports of such cases in the literature. 1 ^,^ 2 Here, the authors describe the case of a patient with intermittent claudication related to entrapment of the anterior tibial artery. 

## CASE REPORT

 The patient was a 33-year-old female with intermittent claudication affecting the right lower limb. She reported that she felt no pain when walking slowly, but that the pain appeared and increased in intensity as she walked at higher velocities, and that these symptoms had worsened over the previous 2 years. She had no comorbidities and was a non-smoker. On physical examination, distal pulses were palpable and symmetrical, but during dorsiflexion maneuvers pedal pulses were attenuated bilaterally, to a greater extent on the right. 

**Figure 1:**
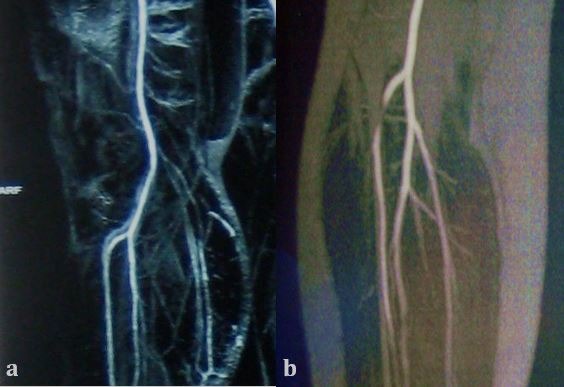
Magnetic resonance angiography showing an absence of compression of the anterior tibial artery at rest (a) compression of the artery during foot dorsiflexion maneuvers (b).

 Doppler ultrasonography of the arteries of the lower limbs was suggestive of extrinsic compression of an artery and magnetic resonance angiography revealed a moderate/accentuated stenosis of the proximal segment of the right anterior tibial artery at the level of the interosseous membrane during dorsiflexion of the feet ( Figure 1 ). 

**Figure 2:**
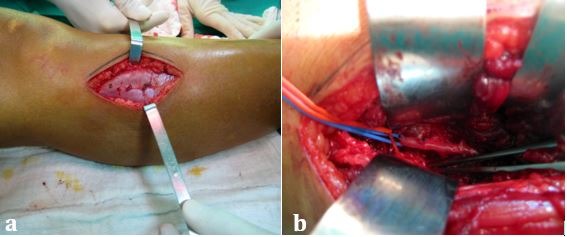
Surgical access via the anterolateral region of the proximal third of the leg (a) and intraoperative appearance after partial resection of the interosseous membrane (b).

 The patient underwent surgical treatment via a longitudinal incision in the anterolateral surface of the right leg, providing access between the tibialis anterior and extensor hallucis longus muscles. The interosseous membrane causing compression and obstruction of the anterior tibial artery was identified and partial resection of the membrane was performed, increasing the size of the opening at the hiatus ( Figure 2 ). 

**Figure 3:**
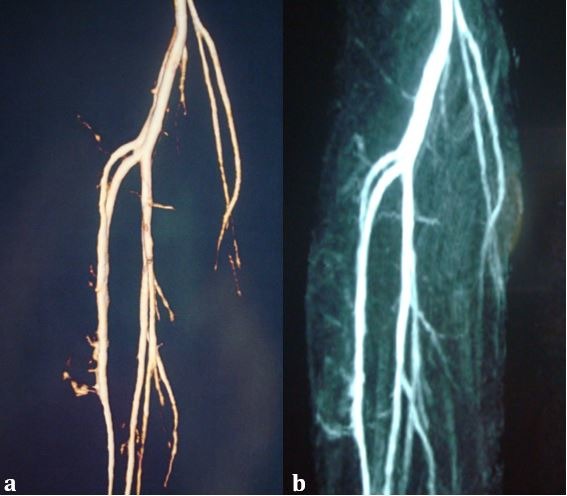
Magnetic resonance angiography after partial resection of the interosseous membrane, showing absence of compression of the anterior tibial artery at rest (a) and during foot dorsiflexion maneuvers (b).

 The patient complained of prolonged postoperative pain, due to hematoma in the anterior compartment caused by manipulation, but recovery progressed at an acceptable rate with physiotherapy. On physical examination, pedal pulses were normal, even during foot dorsiflexion maneuvers. Additionally, magnetic resonance angiography no longer showed compression of the anterior tibial artery ( Figure 3 ). 

## DISCUSSION

 Arterial entrapment syndromes occur when there is an abnormal anatomic relationship between the vessel and the adjacent muscular and tendinous structures, subjecting the artery to compression. Since compressions are generally caused by muscle and tendon structures, they initially manifest during activities that involve use of these structures. 2 ^,^ 3

 Considering vascular compressions in the lower limbs, popliteal artery entrapment syndrome, while uncommon, is widely reported in the literature, and several subtypes have been described. 2 Entrapment of other arteries is rare, and there are only reports of sporadic cases. 4

 In the case of the anterior tibial artery, the artery passes through the interosseous membrane via an oval osteofibrous space to enter the anterior compartment. Along the upper two-thirds of the tibia, the anterior tibial artery runs along the anterior surface of the interosseous membrane, between the anterior tibialis and the extensor hallucis longus muscles. In the lower portion of its path, it is in direct contact with the tibia. Therefore, as a result of its anatomic location, entrapment of the anterior tibial artery is most often related to tibial fractures. 5 ^,^ 6 However, in the case described here, there was no history of any type of trauma or orthopedic intervention involving the limb, nor any type of bone abnormality. Considering the level of the entrapment and the anatomic relationships between the structures in that area, we concluded that the interosseous membrane could be the anatomic structure responsible for entrapment. This theory was only confirmed by partial resection of the interosseous membrane, around the anterior tibial artery, after which there was obvious improvement of the intermittent claudication complaint. Further confirmation that the interosseous membrane was indeed responsible for compression of the artery was provided by magnetic resonance angiography conducted after the treatment, which no longer showed evidence of entrapment, even during flexion maneuvers. 

 In view of this syndrome’s clinical presentation, there are two important differential diagnoses to be considered: chronic compartment syndrome of the anterior compartment of the leg and popliteal artery entrapment syndrome. Popliteal artery entrapment syndrome also presents with intermittent claudication primarily affecting muscle groups in the calf. When compression of the anterior tibial artery is present, restricting flow, involvement of this compartment is more accentuated, and the pain is described as being predominantly in the anterolateral musculature of the leg. 2 This location of pain is the same as described by patients with chronic compartment syndrome of the anterior compartment of the leg. This syndrome generally affects athletes who have hypertrophy of this muscle group, which is restricted in a space of fixed dimensions, causing increased pressure within the compartment, resulting in restricted perfusion and consequent pain. 3 This was an important differential diagnosis that was considered in the case of the patient described here, because of the similarity in clinical presentation. However, we ruled out this hypothesis because of the absence of muscular hypertrophy and the absence of any history of physical activity, so it was not necessary to measure intracompartmental pressure. 

 Entrapment of the anterior tibial artery is a rare cause of atypical lower limb claudication, and some patients remain asymptomatic even when the artery is embolized or traumatized, but this condition should be considered as a possibility in differential diagnosis of young patients. Compression of this vessel by the interosseous membrane has not previously been described in the literature, according to a thorough review of the literature, and this is the first report of the phenomenon. 

## References

[1] Weichman K, Berland T, Mackay B, Mroczek K, Adelman M (2010). Intermittent foot claudication with active dorsiflexion: the seminal case of dorsalis pedis artery entrapment. Ann Vasc Surg.

[2] Tucker AK (2010). Chronic exertional compartment syndrome of the leg. Curr Rev Musculoskelet Med.

[3] Benson RA, Loftus IM (2015). Anterior tibial artery entrapment syndrome: an unusual cause of angiosomal ischaemia. Int J Cardiovasc Res.

[4] Miki RA, Lawrence JP, Gillon TJ, Lawrence BD, Zell RA (2008). Anterior tibial artery and deep peroneal nerve entrapment in spiral distal third tibia fracture. Orthopedics.

[5] Bou S, Day C (2014). Atypical presentation of popliteal artery entrapment syndrome: involvement of the anterior tibial artery. PM R.

[6] Tan ETL, Tan TJ, Poon KB (2016). Entrapment of the deep peroneal nerve and anterior tibial vessels by a spiral tibial fracture causing partial non-union: a case report. Skeletal Radiol.

