# Supplementary material for: Anterior tibial artery entrapment syndrome: case report
Source: J Vasc Bras. 2018 Apr-Jun;17(2):165–9. doi: 10.1590/1677-5449.010017 (PMC6205700; doi:10.1590/1677-5449.010017)
Supplement: Translation

# Translation: Síndrome do aprisionamento da artéria tibial anterior: relato de caso

**Authors:** Marcio Miyamotto, Leandro Castro, Gabrielle Simões Marcusso, Bruna Zimmerman Angelo, Danielle Corrêa de Andrade, Izara Castro de Souza, Ricardo César Rocha Moreira

**Affiliations:** 1 Pontifícia Universidade Católica do Paraná – PUC-PR, Hospital Universitário Cajuru – HUC, Serviço de Cirurgia Vascular e Endovascular, Curitiba, PR, Brasil.; 2 Instituto VESSEL de Aperfeiçoamento Endovascular de Curitiba, Curitiba, PR, Brasil.; 3 Hospital Nossa Senhora das Graças – HNSG, Serviço de Cirurgia Vascular e Endovascular Elias Abrão, Curitiba, PR, Brasil.; 4 Pontifícia Universidade Católica do Paraná – PUC-PR, Hospital Universitário Cajuru – HUC, Liga Acadêmica de Medicina Vascular – LAMEV, Curitiba, PR, Brasil.

**Keywords:** artéria tibial anterior, claudicação intermitente, compressão arterial

## Abstract

A claudicação intermitente é uma queixa geralmente relacionada com oclusão arterial secundária a doença aterosclerótica. Entretanto, em pacientes jovens com queixa de claudicação, outras causas devem ser pesquisadas, como a síndrome do aprisionamento da artéria poplítea, síndrome compartimental crônica, compressões ósseas e arterites. Os autores relatam o caso de um paciente com claudicação intermitente devido à compressão extrínseca da artéria tibial anterior pela membrana interóssea, diagnosticada através de angiorressonância durante manobras de dorsiflexão do pé. A paciente foi tratada com sucesso através da liberação do fator compressivo, evoluindo com melhora dos sintomas.

## INTRODUÇÃO

 A doença obstrutiva periférica de etiologia aterosclerótica é a causa mais comum de claudicação intermitente, sendo responsável por mais de 90% dos casos. Entretanto, em pacientes mais jovens, sem fatores de risco para doença aterosclerótica, é imperativo pesquisar outras causas envolvendo, por exemplo, compressão arterial extrínseca por tecidos moles, como na síndrome do aprisionamento da artéria poplítea, síndrome compartimental crônica, compressões por alterações ósseas e arterites 1 ^-^ 3 . 

 As obstruções não ateroscleróticas geralmente são causadas por compressões relacionadas à síndrome do aprisionamento da artéria poplítea, levando a um quadro típico de claudicação intermitente das pernas, conhecida como claudicação espástica. A ocorrência de aprisionamento de outros vasos da perna é rara, havendo poucos relatos de caso são disponíveis na literatura 1 ^,^ 2 . Os autores relatam o caso de uma paciente com claudicação intermitente relacionada ao aprisionamento da artéria tibial anterior. 

## RELATO DO CASO

 Paciente de 33 anos, sexo feminino, com queixa de claudicação intermitente de membro inferior direito. Referia que a dor era ausente ao caminhar lentamente porém aparecia e piorava de intensidade ao caminhar com maior velocidade, com piora dos sintomas nos últimos dois anos. Não apresentava outras comorbidades e não era tabagista. Ao exame físico, os pulsos distais eram palpáveis e simétricos, porém apresentava diminuição dos pulsos pediosos bilateralmente durante as manobras de dorsiflexão, mais acentuada à direita. 

 O eco-Doppler arterial de membros inferiores sugeriu compressão arterial extrínseca e a angiorressonância evidenciou uma estenose moderada/acentuada no segmento proximal da artéria tibial anterior direita ao nível da membrana interóssea, com a dorsiflexão dos pés ( Figura 1 ). 

**Figura 1:** Angiorressonância evidenciando ausência de compressão da artéria tibial anterior ao repouso (a) e compressão da artéria durante as manobras de dorsiflexão do pé (b).

 A paciente foi submetida a tratamento cirúrgico através de incisão longitudinal em face anterolateral da perna direita e acesso entre os músculos tibial anterior e extensor longo do hálux. Foi identificada a membrana interóssea que causou a compressão e a obstrução da artéria tibial anterior e foi realizada a ressecção parcial da membrana, aumentando a abertura do hiato ( Figura 2 ). 

**Figura 2:** Acesso cirúrgico em região anterolateral do terço proximal de perna (a) e aspecto cirúrgico após ressecção parcial da membrana interóssea (b).

 Na evolução, a paciente apresentou dor pós-operatória mais prolongada devido a hematoma no compartimento anterior pela manipulação, apresentando recuperação adequada após acompanhamento fisioterápico. Ao exame físico, os pulsos pediosos apresentavam pulsatilidade normal, mesmo durante as manobras de dorsiflexão do pé. Da mesma forma, a angiorressonância não demonstrou compressão da artéria tibial anterior ( Figura 3 ). 

**Figura 3:** Angiorressonância após a ressecção parcial da membrana interóssea evidenciando ausência de compressão da artéria tibial anterior ao repouso (a) e durante as manobras de dorsiflexão do pé (b).

## DISCUSSÃO

 As síndromes do aprisionamento arterial ocorrem quando existe uma relação anatômica anormal entre o vaso e as estruturas musculotendinosas adjacentes, causando compressão da artéria. Como as compressões geralmente ocorrem por estruturas musculares e tendíneas, inicialmente as compressões ocorrem geralmente na realização de atividades que envolvem a utilização dessas estruturas 2 ^,^ 3 . 

 Considerando as compressões vasculares nos membros inferiores, a síndrome do aprisionamento da artéria poplítea, apesar de incomum, é amplamente descrita na literatura, sendo descritos vários subtipos 2 . O aprisionamento de outras artérias é raro, existindo apenas relatos de casos esporádicos 4 . 

 No caso da artéria tibial anterior, a artéria atravessa a membrana interóssea por um espaço oval osteofibroso para penetrar no compartimento anterior. Nos dois terços superiores da tíbia, a artéria tibial anterior situa-se sobre a superfície anterior da membrana interóssea entre os músculos tibial anterior e extensor longo do hálux. Na porção inferior do seu trajeto, relaciona-se diretamente com a tíbia. Portanto, devido à sua localização anatômica, o aprisionamento da artéria tibial anterior está mais relacionado às fraturas da tíbia 5 ^,^ 6 . No caso descrito, não havia história de qualquer tipo de trauma ou intervenção ortopédica no referido membro, bem como qualquer anormalidade óssea. Considerando o nível do aprisionamento e as relações anatômicas das estruturas da região, concluímos que a membrana interóssea poderia ser a estrutura anatômica responsável pelo aprisionamento. Essa teoria foi confirmada apenas após a ressecção parcial da membrana interóssea, ao redor da artéria tibial anterior, após a qual houve uma evidente melhora da queixa de claudicação intermitente. Outra confirmação de que a membrana interóssea era realmente responsável pela compressão arterial foi a angiorressonância realizada após o tratamento, a qual não evidenciava mais o aprisionamento, mesmo durante a realização das manobras de flexão. 

 Considerando a apresentação clínica dessa síndrome, devemos considerar dois importantes diagnósticos diferencias: a síndrome compartimental crônica do compartimento anterior da perna e a síndrome do aprisionamento da artéria poplítea. A síndrome do aprisionamento da artéria poplítea também se apresenta com quadro de claudicação intermitente acometendo principalmente os grupos musculares da panturrilha. Quando existe compressão da artéria tibial anterior com restrição de fluxo, o acometimento desse compartimento é mais acentuado, sendo que a dor é relatada como sendo predominante na musculatura anterolateral da perna 2 . Essa localização é a mesma da dor relacionada a síndrome compartimental crônica do compartimento anterior da perna. Essa síndrome geralmente ocorre em atletas com hipertrofia desse grupo muscular, que fica restrita num espaço de dimensões fixas e pode levar a um aumento da pressão dentro do compartimento, resultando em restrição de perfusão e consequente dor 3 . Esse foi um importante diagnóstico diferencial considerado no caso dessa paciente, devido à semelhança na apresentação clínica. Porém, a ausência de hipertrofia muscular, bem como a ausência de histórico de atividade física, fizeram com que descartássemos essa hipótese, não sendo necessária, portanto, a aferição da pressão intracompartimental. 

 O aprisionamento da artéria tibial anterior é uma rara causa de claudicação atípica de membro inferior, sendo que alguns pacientes permanecem assintomáticos mesmo quando essa artéria é embolizada ou traumatizada, mas deve ser considerada como um diagnóstico diferencial em pacientes jovens. A compressão desse vaso pela membrana interóssea não foi publicada previamente na literatura, de acordo com nossa ampla revisão, sendo este o primeiro relato de tal fenômeno.
